# The complexity of NF-κB signaling in inflammation and cancer

**DOI:** 10.1186/1476-4598-12-86

**Published:** 2013-08-02

**Authors:** Bastian Hoesel, Johannes A Schmid

**Affiliations:** 1 Department of Vascular Biology and Thrombosis Research, Center for Physiology and Pharmacology, Medical University Vienna, Schwarzspanierstraße 17, 1090 Vienna, Austria

**Keywords:** NF-kappa B signaling, Cancer, Inflammation, Cooperativity, Crosstalk

## Abstract

The NF-κB family of transcription factors has an essential role in inflammation and innate immunity. Furthermore, NF-κB is increasingly recognized as a crucial player in many steps of cancer initiation and progression. During these latter processes NF-κB cooperates with multiple other signaling molecules and pathways. Prominent nodes of crosstalk are mediated by other transcription factors such as STAT3 and p53 or the ETS related gene ERG. These transcription factors either directly interact with NF-κB subunits or affect NF-κB target genes. Crosstalk can also occur through different kinases, such as GSK3-β, p38, or PI3K, which modulate NF-κB transcriptional activity or affect upstream signaling pathways. Other classes of molecules that act as nodes of crosstalk are reactive oxygen species and miRNAs. In this review, we provide an overview of the most relevant modes of crosstalk and cooperativity between NF-κB and other signaling molecules during inflammation and cancer.

## Introduction

**Figure 1:**
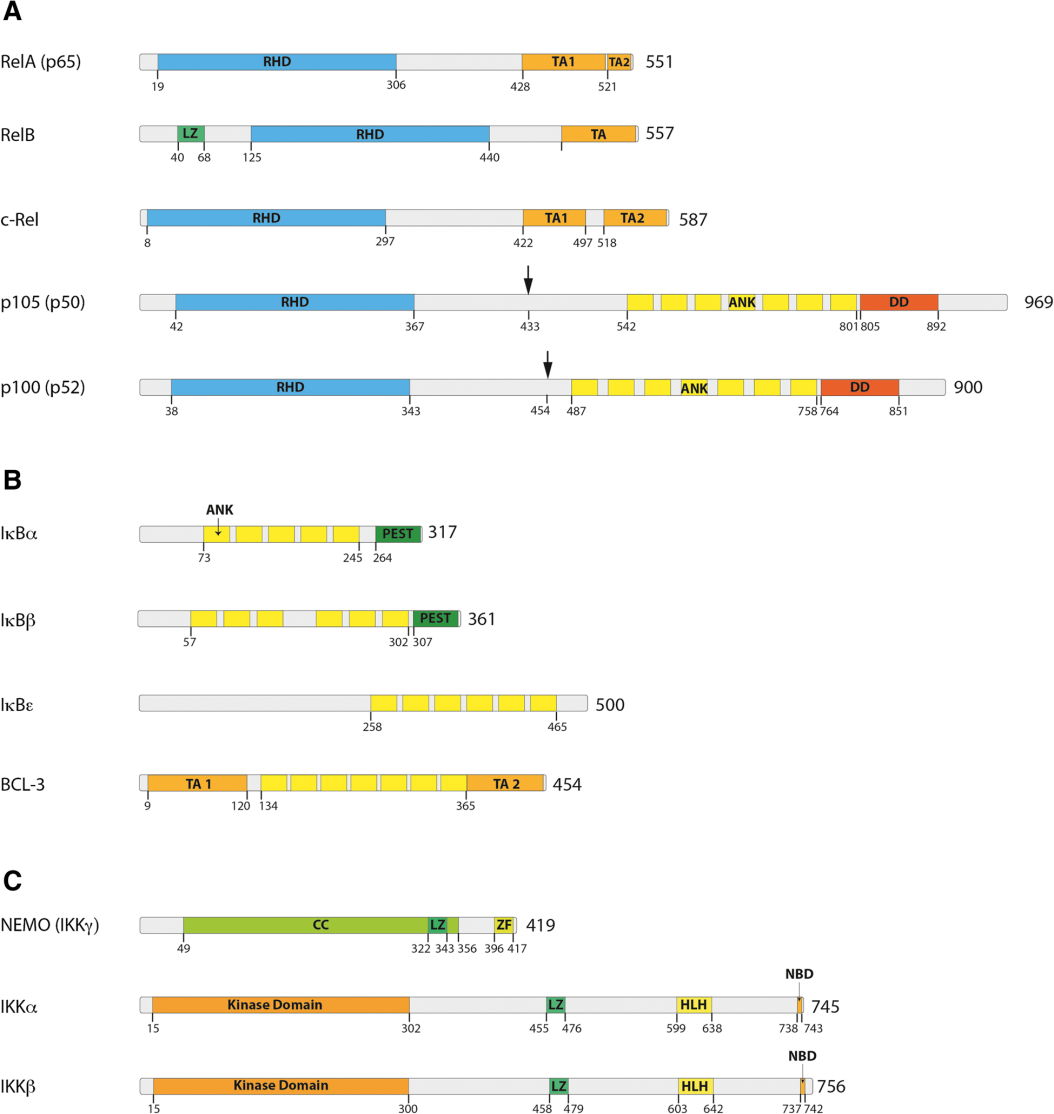
**Members of the NF-κB signaling pathway and the IκB kinase-complex. (A)** The five members of the NF-κB family of proteins: RelA (p65), RelB, c-Rel,NF-κB1 (p105), and NF-κB2 (p100). p105 and p100 are processed to their shorter forms p50 and p52, respectively. All members of the NF-κB family harbor an N-terminal Rel homology domain (RHD), which mediates DNA contact and homo- and heterodimerization. Three family members (RelA, RelB and c-Rel) contain C-terminal transactivation domains (TAs), which are essential for transcriptional activity. **(B)** The IκB family of proteins consists of four members: IκBα, IκBβ, IκBϵ and BCL-3. These proteins are characterized by the presence of ankyrin (ANK) repeats, which mediate binding of IκBs to the NF-κB family of proteins. Based on the presence of ankyrin repeats, p100 and p105 can also be included into the IκB family – as their DNA-binding RHD domain is covalently linked to an IκB-like inhibitory domain. In addition to the ANK repeats IκBα and IκBβ contain PEST domains, which are enriched in proline, glutamate, serine and threonine and are required for constitutive turnover. BCL-3 differs from other IκB family members by containing TA domains, which mediate transcriptional activity when BCL-3 is associated with NF-κB dimers that bind to DNA. **(C)** The three most important members of IκB kinase (IKK) complex: NF-κB Essential Modulator (NEMO or IKKγ), IκB kinase α, (IKKα or IKK1) and IκB kinase β (IKKβ or IKK2). Further abbreviations: leucin-zipper-like motif (LZ), death domain (DD), coiled-coil domain (CC), zinc-finger domain (ZF), helix-loop-helix domain (HLH), NEMO-binding domain (NBD). It is important to note that the total number of amino acids of protein as well as the start and end of some domains can differ between publications and databases.

The transcription factor NF-κB was discovered in 1986 as a nuclear factor that binds to the enhancer element of the immunoglobulin kappa light-chain of activated B cells (thereby coining the abbreviation NF-κB) [1]. Soon afterwards it became clear that proteins, which harbor this specific DNA binding activity are expressed in nearly all cell types and regulate many target genes with a whole variety of functions [2]. In total, five members of this transcription factor family have been identified, designated as p65 (RelA), RelB, c-Rel, NF-κB1 and NF-κB2 (Figure 1A). In contrast to the other family members, NF-κB1 and NF-κB2 are synthesized as pro-forms (p105 and p100) and are proteolytically processed to p50 and p52 (Figure 1A, black arrows), respectively [3]. All five members of this protein family form homo- or heterodimers and share some structural features, including a Rel homology domain (RHD), which is essential for dimerization as well as binding to cognate DNA elements [4]. In most quiescent cells these dimers are bound to inhibitory molecules of the IκB family of proteins (inhibitors of NF-κB) (Figure 1B). These inhibitors are characterized by ankyrin repeats, which associate with the DNA-binding domains of the transcription factors thereby making them transcriptionally inactive. Interestingly, p105 and p100, the precursors of p50 and p52, also contain ankyrin repeats, which are cleaved upon maturation - thus comprising their own internal inhibitors. In contrast to the other members of the NF-κB family these two proteins do not contain a transactivation domain [5]. As a consequence, dimers of p50 and p52, which bind to NF-κB elements of gene promoters, act as transcriptional repressors [6]. However, when p50 or p52 are bound to a member containing a transactivation domain, such as p65 or RelB, they constitute a transcriptional activator. Another interesting aspect is that one member of the IκB family, Bcl-3, also contains transactivation domains (Figure 1B) and can bind to dimers of p50 and p52 rendering the complex transcriptionally active [7-9]. The complexity of this transcriptional regulation system is also augmented by the fact that different NF-κB dimers have differential preferences for variations of the DNA-binding sequence [10]. Thus different target genes are differentially induced by distinct NF-κB dimers. Furthermore, NF-κB subunits also contain sites for phosphorylations and other post-translational modifications which are important for activation and crosstalk with other signaling pathways [11]. Binding of NF-κB dimers to IκB molecules does not only prevent binding to DNA, but also shifts the steady-state localization of the complex to the cytosol. Nevertheless, shuttling between cytosol and nucleus does occur [12,13], which might be a basis for a low basal transcriptional activity of NF-κB given that the IκB/NF-κB complex is subject to dissociation and re-association processes.

### Signaling pathways activating NF-κB

In general, activation of NF-κB occurs by release from the IκB molecules or by cleavage of the inhibitory ankyrin repeat domains of p100 and p105. This is achieved by proteasomal degradation of the inhibitors or by partial degradation of the precursors. A prerequisite for the degradation is polyubiquitination of the target molecules with lysine-48 linked ubiquitin chains, which is catalyzed by SCF^βTrCP^ type E3-ligases. These ubiquitination enzymes require a specific double phosphorylation on the substrate as recognition site. The latter is catalyzed by an enzyme complex containing IκB kinases (IKK1/IKKα and IKK2/IKKβ) and at least one non-catalytic accessory protein (NF-κB Essential Modulator, NEMO or also termed IKKγ) [14-16]. This IκB kinase or IKK-complex binds to additional components and interacts with upstream signaling molecules and kinases. A great variety of stimuli can activate the IKK complex by different mechanisms including phosphorylation of the activation loop of IKK’s (S177 and S181 of IKK2, [17]) by upstream kinases or via proximity-induced self-activation of IKK-dimers by mutual trans-phosphorylation [18]. Kinases that mediate phosphorylation and activation of IKKs include the NF-κB inducing kinase NIK, which predominantly phosphorylates IKK1 on S176 [19,20], as well as MEKK1, MEKK2, MEKK3 and TGF- β –activating kinase 1 (TAK1) [21-23]. TAK1 is a member of a larger protein kinase complex, which consists of TAK1, TAB1 and TAB2 and can phosphorylate IKK2 as well as NIK [23,24]. MEKK3 is a member of the MAP3K family and is known to have a role in TLR4 mediated signaling [25]. Furthermore it seems that TAK1 and MEKK3 have differential roles in interleukin and Toll-like receptor mediated NF-κB activation [25,26]. Lysine-63 linked polyubiquitin chains attached to signaling molecules by RING-type E3 ligases (such as TRAF2 or TRAF6) have been demonstrated to act as activation platform [27,28], as well as linear polyubiquitination of upstream effector molecules [29,30]. These various activation mechanisms guarantee that all different stress situations can induce the catalytic activity of IKKs thereby leading to the liberation and activation of the general stress response factor NF-κB. In addition, they provide a basis for manifold crosstalk with other signaling pathways, as well as complex feedback circuits allowing for a fine-tuning of the response. Since K63-linked or linear polyubiquitination provide mechanisms for activation of the NF-κB pathway it is plausible that several of the feedback inhibitors represent de-ubiquitinases (DUB’s) such as A20 [31] or CYLD [32].

**Figure 2:**
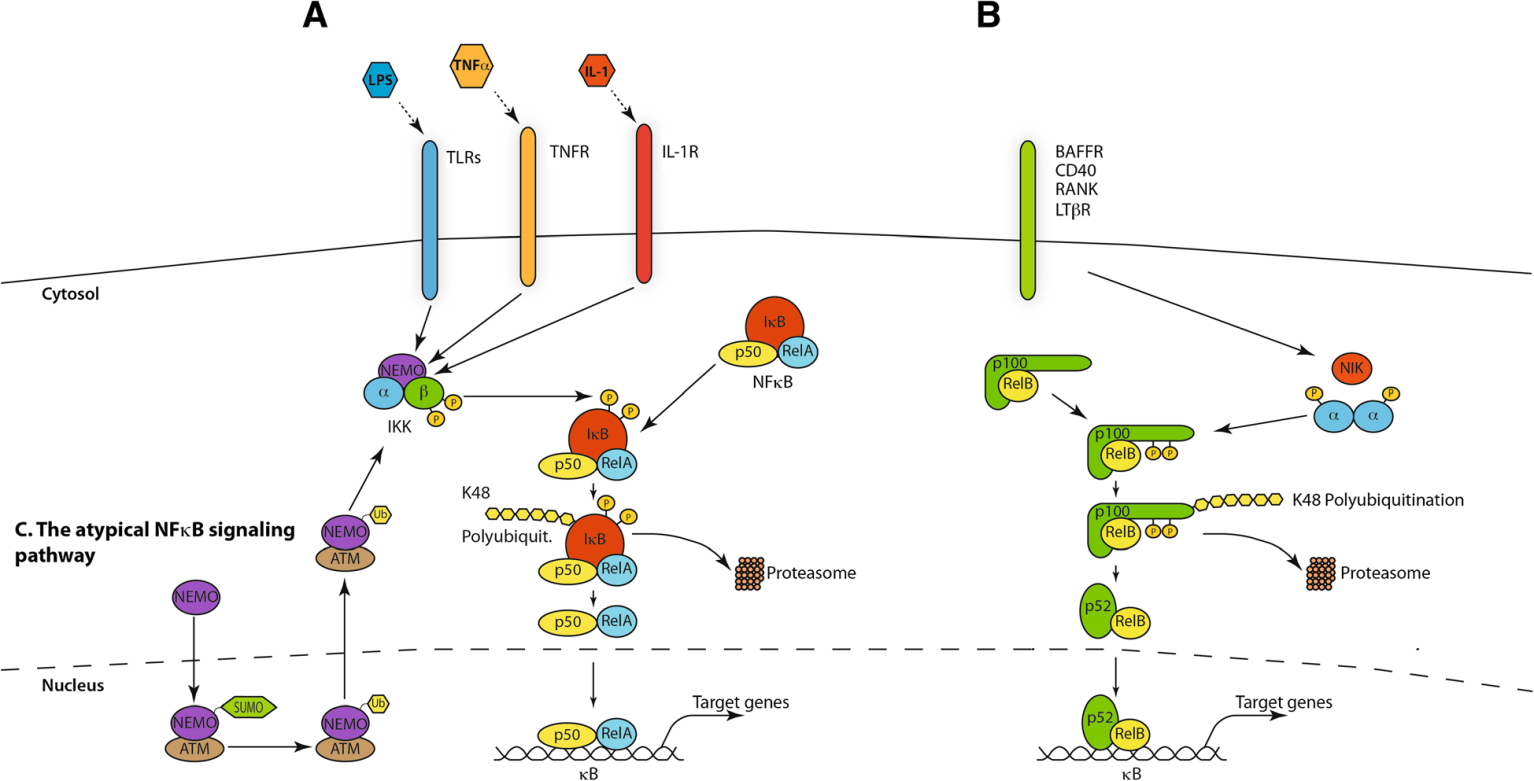
**The canonical, non-canonical and the atypical NF-κB signaling pathway. (A)** In the canonical NF-κB signaling pathway lipopolysaccharides (LPS), tumor necrosis factor α (TNFα) orinterleukin-1 (IL-1) activate Toll-like receptors (TLRs), tumor necrosis factor receptor (TNFR) and interleukin-1 receptor (IL-1R), respectively. Through a variety of adapter proteins and signaling kinases this leads to an activation of IKKβ in the IKK complex, which can then phosphorylate IκBα on Serine residues S32 and S36. This phosphorylation is a prerequisite for its subsequent polyubiquitination, which in turn results in proteasomal degradation of IκBα. NF-κB homo- or heterodimers can then translocate to nucleus and activate target gene transcription. **(B)** In the non-canonical NF-κB signaling pathway, activation of B-cell activation factor (BAFFR), CD40, receptor activator for nuclear factor kappa B (RANK) or lymphtoxin β-receptor (LTβR), leads to activation of IKKα by the NF-κB-inducing kinase (NIK). IKKα can the phosphorylate p100 on serine residues S866 and S870. This phosphorylation leads to polyubiquitination of p100 and its subsequent proteasomal processing to p52.p52-RelB heterodimers can then activate transcription of target genes. **(C)** In the atypical NF-κB signaling pathway, genotoxic stress leads to a translocation of NEMO to the nucleus where it is sumoylated and subsequently ubiquitinated. This process is mediated by the ataxia telangiectasia mutated (ATM) checkpoint kinase. NEMO and ATM can then return to the cytosol where they activate IKKβ.

In the canonical activation pathway (Figure 2A), excitatory signaling can be mediated through Toll-like receptors (TLRs), Interleukin-1 receptor (IL-1R), tumor necrosis factor receptor (TNFR) and antigen receptors. Typical stimulating signaling molecules are tumor necrosis factor α (TNFα), lipopolysaccharides (LPS), which are bacterial cell wall components, and interleukin-1 β (IL-1β) [18,33]. Stimulation through these receptors leads to activation of the IκB kinase (IKK) complex, which in turn phosphorylates IκBα primarily by IKK2.

An alternative pathway of NF-κB activation, also designated as non-canonical pathway [34] (Figure 2B) originates from different classes of receptors including B-cell activation factor (BAFFR), lymphotoxin β-receptor (LTβR), CD40, receptor activator for nuclear factor kappa B (RANK), TNFR2 and Fn14 [35]. These lead to activation of the NF-κB inducing kinase NIK, which phosphorylates and activates predominantly IKK1. The activity of the latter enzyme induces phosphorylation of p100 resulting in its ubiquitination and partial degradation to p52 [36]. The mechanisms leading to activation of the non-canonical pathway are thus independent of the activity of IKK2 and NEMO [37].

In many cases, p100 is associated with RelB, so that its proteolytic processing induces the formation of a transcriptionally active RelB/p52-complex [38,39]. Besides the canonical and the alternative pathway, additional pathways of NF-κB activation exist, sometimes termed atypical activation pathways (Figure 2C). One of these is activation of the IKK complex after genotoxic stress via the kinase ATM leading to ubiquitination of NEMO [40]. Others involve tyrosine kinases or casein kinase 2 [41-43]. The epidermal growth factor receptor (EGFR) tyrosine kinase has for example been shown to promote NF-κB dependent transcription in ovarian cancer [44].

**Figure 3:**
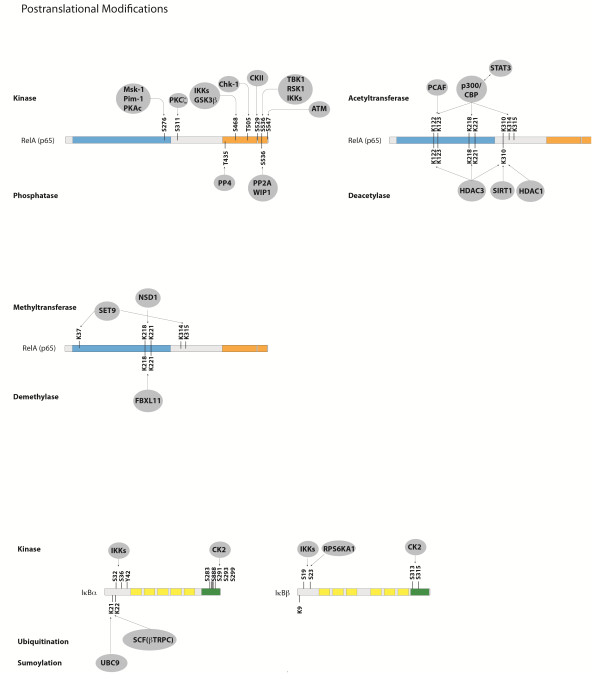
**Post-translational modifications of RelA, IκBα and IκBβ.** Phosphorylations, acetylations and methylations of RelA are shown, as well as phosphorylations, ubiquitination and sumoylation of IkBα and IkB.

**Figure 4:**
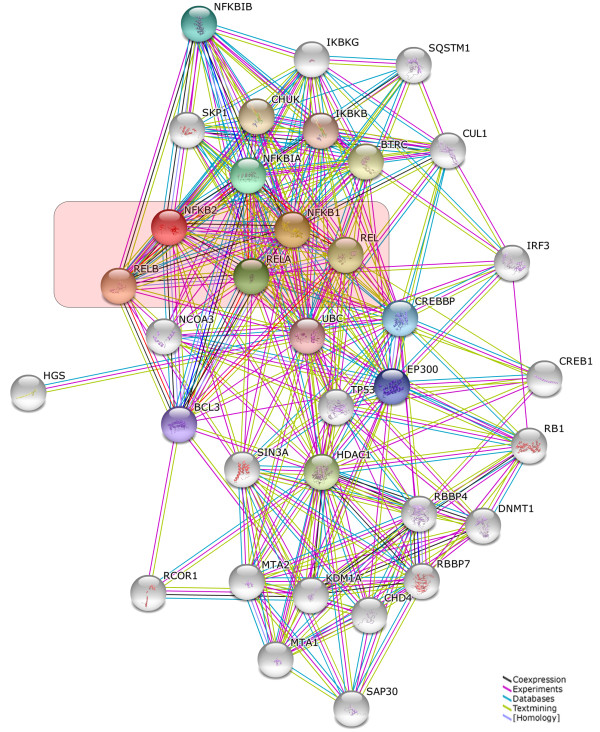
**Network of NF-κB interactors.** Evidence view of the STRING database output depicting functional and physical interactors of the NF-κB proteins, RelA, Rel (c-Rel), RelB, NFKB1 and NFKB2 obtained from: http://string-db.org/. The five NF-κB proteins are highlighted in red.

After the liberation of various NF-κB dimers following activation of IKKs, their steady state localization is normally shifted to the nucleus and the Rel Homology Domains are free to bind cognate DNA-sequences in the enhancer elements of target gene promoters. Depending on the accessibility of the genome regulated by epigenetic mechanisms and the cell type, thousands of different target genes can be transcriptionally activated. This activation is further controlled by additional transcription factors, which can either enhance or reduce the effect of NF-κB – establishing another level of complexity and crosstalk with signaling pathways that activate other transcription factors. The manifold post-translation modifications of RelA add another layer of complexity to NF-κB signaling. These have been shown to be necessary for various aspects of RelA functions (Figure 3). Amongst these, phosphorylations at specific serine or threonine residue are known to be particularly important since they often stimulate transcriptional activity. The most important phosphorylations of RelA, their positions and the corresponding kinases are summarized in Table 1. The activity of NF-κB is not only influenced by a variety of phosphorylations, but also by dynamic and complex protein-protein interactions generating a sophisticated network of interdependencies and feedback loops [45]. Besides the very well defined interactions between the various members of the NF-κB family, and the interactions with their inhibitors such as IκBα, IκBβ or IκBϵ [46-49], NF-κB molecules have been shown to interact with upstream kinases, with chromatin-modifiers such as histone deacetylases (HDACs), p300 or CBP and also with other transcription factors [50-53]. The network of protein interactions involving NF-κB molecules is very complex. The interaction database *IntAct* (http://www.ebi.ac.uk/intact/) currently lists 306 binary interactions for the NF-κB member RelA alone. To illustrate at least part of this interaction network graphically, we performed a STRING database search (at http://string-db.org/) for proteins interacting either physically or functionally with NF-κB molecules using all five family members as input (Figure 4).

**Table 1:** Positions of Phosphorylations of RelA and corresponding kinases

**Kinase**	**p65 target residue**	**Effect of phosphate**	**References**
**unknown**	**S205**	**stimulates transcriptional activity**	[54]
**MSK1**	**S276**	**stimulates transcriptional activity**	[55]
**PIM1**	**S276**	**stimulates transcriptional activity**	[56]
**PKAc**	**S276**	**stimulates transcriptional activity**	[57,58]
**unknown**	**S281**	**stimulates transcriptional activity**	[54]
**PKCζ**	**S311**	**stimulates transcriptional activity**	[59]
**GSK-3β**	**S468**	**stimulates transcriptional activity**	[60]
**IKK2**	**S468; S536**	**stimulates transcriptional activity and nuclear import**	[61,62]
**IKKϵ**	**S468;S536**	**stimulates transcriptional activity**	[63,64]
**CKII**	**S529**	**stimulates transcriptional activity**	[65]
**CaMKIV**	**S535**	**stimulates transcriptional activity**	[66]
**TBK1**	**S536**	**stimulates transcriptional activity**	[67]
**IKK1**	**S536**	**stimulates transcriptional activity and stabilization**	[68]
**RSK1**	**S536**	**decreases IκBα -mediated nuclear export**	[69]
**ATM**	**S547**	**Increased expression of specific genes**	[70]
**unknown**	**T254**	**stabilization and nuclear localization**	[71]
**unknown**	**T435**	**stimulates transcriptional activity**	[72]
**CHK1**	**T505**	**pro-apoptotic effect**	[73]

Termination of the transcriptional activity of NF-κB is mainly achieved by the fact that NF-κB up-regulates its own inhibitors of the IκB family, where the best studied example is IκBα [74,75]. Newly synthesized IκBα enters the nucleus, removes NF-κB from the DNA and relocates it to the cytosol [11]. In addition, negative regulators of the NF-κB signaling pathway such as A20 [31] and CYLD [32] are up-regulated by NF-κB. In acute inflammation, these negative feedback loops usually result in complete de-activation of NF-κB to the normal background level. However, in chronic inflammatory conditions, the persistent presence of NF-κB activating stimuli seems to outperform the inhibitory feedback circuits leading to an elevated constitutive activity of NF-κB.

### The NF-κB signaling pathway in inflammation and cancer

Inflammation is the process of innate immunity in response to physical, physiological and/or oxidative stress and is associated with activation of the canonical NF-κB signaling pathway, which is conserved in all multicellular animals [76]. Inflammation in general and NF-κB in particular have a double-edged role in cancer. On one hand, activation of NF-κB is part of the immune defense, which targets and eliminates transformed cells. This seems to be particularly true for acute inflammatory processes, where full activation of NF-κB is accompanied by a high activity of cytotoxic immune cells against cancer cells [77]. On the other hand, NF-κB is constitutively activated in many types of cancer and can exert a variety of pro-tumorigenic functions. The effectiveness of the immune system against malignant cells has been unveiled by the observation that pharmacologically immune-suppressed individuals, e.g. after organ transplantations, have a higher cancer risk. This anti-tumorigenic function of the immune systems with NF-κB being an important effector of it, has been designated as tumor-immunosurveillance [78]. This immune defense against cancer cells, however, is normally not tight enough to eliminate all the aberrant cells, resulting in a shift to an equilibrium phase, which is often followed by an “escape” phase of the cancer cells, in which they outperform the immune system [79]. The latter two phases seem to be characterized by a chronic inflammatory condition with often only moderately elevated levels of NF-κB activity. The notion that such a constitutive activity of NF-κB exerts a pro-tumorigenic effect is underscored by the observation that patients with chronic inflammatory diseases have higher risks for cancer similar to immune-suppressed patients (see accompanying article). NF-κB activation usually results in the up-regulation of anti-apoptotic genes thereby providing cell survival mechanism to withstand the physiological stress that triggered the inflammatory response. Furthermore, NF-κB induces cytokines that regulate the immune response (such as TNFα, IL-1, IL-6 and IL-8), as well as adhesion molecules, which lead to the recruitment of leukocytes to sites of inflammation. In addition to its role in innate immunity NF-κB signaling was shown to control a great variety of other well conserved cellular processes, including cell proliferation [80,81] and apoptosis [82]. The contribution of inflammation in general and NF-κB in particular to cancer initiation and progression is manifold and complex. It is postulated that the innate immune response of neutrophils that release reactive oxygen species to kill invading pathogens might cause DNA-damage and thus genetic mutations as side effects, thereby triggering tumor initiation [83]. Moreover, NF-κB signaling was shown to contribute to cancer progression by controlling epithelial to mesenchymal transition and metastasis [84]. The latter is often associated with an up-regulation of matrix metalloproteinases (MMPs), loosening the extracellular matrix for an evasion of cancer cells. Finally, NF-κB can also contribute to tumor progression by controlling vascularization of tumors via upregulation of VEGF (vascular endothelial growth factor) and its receptors [85,86].

A tumor can principally establish elevated NF-κB activity by intrinsic or extrinsic factors [87]. On the one hand, enhanced NF-κB activity can be directly induced by mutations of NF-κB genes and/or oncogenes that activate the NF-κB signaling pathway. On the other hand, a tumor can achieve elevated NF-κB activity through increased cytokine release from the tumor microenvironment [76].

Direct mutations of NF-κB signaling genes have so far been detected mainly in lymphoid malignancies. Amplification and point mutations of RelA were detected in human B-cell lymphomas such as Hodgkin lymphoma and to a lower extent also in T-cell lymphomas, reflecting the direct oncogenic potential of the NF-κB signaling pathway which was suggested since the initial discovery of the oncogenic RelA homologue v-Rel [88]. Furthermore, chromosomal truncations of the NFKB2 gene have been detected in certain lymphomas [89,90]. Moreover, mutations of other members of the NF-kB signaling pathway including Bcl-3 and c-Rel have been detected in B-cell leukemia and several types of B-cell lymphomas, respectively [91-94].

In solid tumors, in contrast, direct mutations of the NF-κB signaling pathway are rare events [95]. Nevertheless, they do occur, as exemplified by a recently discovered gene fusion between IKK2 and TNPO1 (transportin 1), which resulted in elevated IKK2 expression levels in prostate cancer [96]. Furthermore, a recent study on breast cancer revealed mutations in NFKB1, the upstream kinase IKK2, as well as the inhibitors IκBα and IκBϵ [97]. Studies with transgenic mice suggest a direct contribution of the NF-κB signaling pathway to the development of various solid tumors. The maybe best-studied example is inflammation associated colon cancer, where IKK2-induced NF-κB within intestinal epithelial cells has an essential role for tumor formation. Furthermore, IKK2-mediated NF-κB activity within myeloid cells of the tumor environment contributes to tumor progression by inducing the secretion of cytokines and growth factors [98]. A different inflammation-associated type of cancer is hepatocellular carcinoma, a form of liver cancer, which can occur after viral hepatitis or after liver damage induced by carcinogenic substances. Interestingly, the role of NF-κB in liver cancer seems to depend very much on the precise mechanism of cancer development. Tumors associated with chronic inflammation seem to require NF-κB within hepatocytes as anti-apoptotic survival factor. However, in certain types of chemically induced liver cancer, hepatocytic NF-κB in contrary acts as tumor suppressor as shown by mouse studies with hepatocyte-specific deletion of IKK2 or NEMO and treatment with diethylnitrosamine (DEN) as carcinogen [99]. Nevertheless, this type of cancer still requires NF-κB within Kupffer cells (the resident macrophages of the liver), which is essential for secretion of IL-6 and activation of STAT3 in neighboring hepatocytes. Thus, the cellular location of NF-κB activity is fundamental for the development of liver cancer. Another cancer that depends on NF-κB activity is melanoma, as it could be shown that HRas-mediated initiation of tumorigenesis requires IKK2-mediated NF-κB activation in a mouse model of melanoma [100] and even for lung cancer it could be demonstrated that IKK2 and NF-κB are crucial cofactors [101]. In general, aberrant NF-κB activity seems to have an important role as co-factor in solid tumors by acting as survival factor for transformed cells, which would otherwise become apoptotic or senescent. This elevated constitutive NF-κB activity is usually achieved through continuous release of cytokines by macrophages in the tumor microenvironment. However, there is still a mystery in the crosstalk between solid tumors and neighboring macrophages: While most tumors are characterized by elevated levels of cytokines released by classically (M1)-activated macrophages, such as TNFα and IL-1β, the macrophages in the tumor microenvironment seem to switch to the M2-phenotype, the so-called alternatively activated form, which seems to be the predominant form of tumor-associated macrophages (TAMs) - releasing rather anti-inflammatory cytokines. IKK2 and NF-κB apparently polarize macrophages towards the alternatively activated M2 phenotype, which tolerates and even fosters the tumor instead of attacking it [102]. It seems possible, that the timing is crucial for the crosstalk between tumor cells and macrophages. An initial inflammatory environment triggered by the tumor might induce the secretion of TNFα and IL-1β from resident macrophages, whereas prolonged tumor growth and chronic inflammation might lead to a shift towards tumor-associated, M2-type macrophages – a notion that is also in line with the concept that tumors are interpreted by the organism often as “wounds that do not heal” [103]. In addition to the role of NF-κB for survival of cancer cells or the response of immune cells to cancer, NF-κB has recently been shown to be activated in cancer stem cells (CSCs), where it can promote a pro-inflammatory environment, inhibit apoptosis and stimulate cell-proliferation. Cancer stem cells comprise only a minor subpopulation of cancer cells and are thought to mediate tumor growth and resistance to chemotherapy [104,105].

### Crosstalk of NF-κB with other transcription factors

In general, transcription factors can exert mutual influences by a variety of mechanisms. One of them is direct physical association, which can influence transcriptional activity or DNA-binding. Direct binding to NF-κB is known for several transcription factors including STAT3, p53, estrogen receptor, ATF3, SMAD3 and 4 (according to the IntAct database). Furthermore, transcription factors often bind in close vicinity to each other on their cognate sequences of promoter or enhancer elements thereby facilitating the recruitment of components of the transcriptional machinery. In this way, they can either enhance or repress the function of another transcription factor [106]. Functional links between NF-κB and other transcription factors are therefore manifold – depending on the promoter structure of target genes. Several of these links have been studied and characterized in more detail, in particular those with STAT family members (signal transducers and activators of transcription) and with the p53 tumor suppressor [107,108].

NF-κB and STAT3 cooperatively regulate a number of target genes including anti-apoptotic as well cycle control genes. Moreover, they also synergistically control a common set of genes encoding for cytokines and chemokines [109,110]. It has been shown that p65 and p50 NF-κB interact physically with STAT3, facilitating NF-κB recruitment to STAT3 promoters and vice versa. In addition, there is another level of regulation, as it has been shown that STAT3 can modify RelA post-translationally by recruitment of the acetyltransferase p300, mediating acetylation of NF-κB and prolongation of its nuclear retention [111]. By that means STAT3-mediated acetylation affects NF-κB activity - a mechanism, which plays a role in cancer – as it is often the result of chronic stimulation with cytokines in a tumor microenvironment. This chronic inflammation results in an elevated constitutive activity of NF-κB and a release of cytokines such as IL-6, which by itself activates STAT3. This can then further prolong NF-κB activity via enhancing its acetylation. However, STAT3 can also have a tumor suppressor function as shown recently for intestinal cancer, where it affects the activity of other members of the STAT family and the expression of cell adhesion molecules [112]. While there is in most cases a positive feedback between NF-κB and STAT3, a mutual inhibition has been reported for NF-κB and p53 [113]. The NF-κB subunit RelA has been shown to inhibit p53 dependent transactivation, while p53 can also suppress NF-κB transcriptional activity [113]. Recent data indicates that mutant p53 elevates expression of p52 NF-κB by inducing acetylation of histones via recruitment of CBP and Stat2 on its promoter via CBP-mediated acetylation [114]. Moreover, it has been shown that the crosstalk between p53 and NF-κB might also be necessary for full activity of NF-κB after certain types of stimulation even including TNFα. This positive cooperativity with NF-κB seems to be stronger for p53 mutants, providing a potential explanation for the fact that p53 mutants are much more frequently observed in cancer than p53 deletions [115]. More recently, crosstalk of NF-κB with another transcription factor involved in certain types of cancer has been identified – that with the Ets family member ERG.

ERG has a role in various leukemia [116,117] and in Ewing sarcoma [118], and more recently it has been found as being overexpressed in some prostate cancer patients due to a genomic fusion with the androgen dependent promoter of the TMPRSS2 gene [119]. Interestingly, an increased NF-κB activity was detected particularly in ERG fusion-positive prostate cancer patients and cancer cell lines. It could be shown that the increased NF-κB activity is associated with phosphorylation of p65 on Ser536 involving signaling through TLR4 [120]. ERG also appears to regulate expression of the NF-κB target gene ICAM-1 in endothelial cells [121] and additional data suggest that ERG can stimulate the CXR4/CXCL12 axis, which contributes to metastasis [122].

### Crosstalk of the NF-κB pathway with other signaling pathways

While our picture of signaling is often rather a one-dimensional scheme of a signal cascade it is becoming clear that biological signaling is better described by a dynamic signaling network with complex feedback circuits. However, it is difficult to show this reality of signaling in a printed manner – as the only way to depict that in publications is by drawing connections and arrows between signaling pathways and molecules. There are numerous interactions, links and cooperativities between the NF-κB pathway and other signaling pathways. One example is the influence of Glycogen Synthase Kinase GSK3-β on NF-κB signaling. GSK-3β is a serine/threonine kinase, which was initially identified as a key regulator of insulin dependent glycogen synthesis [123], and is known to be a mediator of a number of major signaling pathways including the phosphatidyl-inositol-3-kinase (PI3K) pathway, the Wnt pathway, Hedgehog signaling and Notch [124]. It could be shown that GSK-3β has a modulating role for the NF-κB signaling pathway, as it seems to facilitate NF-κB function through post-transcriptional regulation of the NF-κB complex [125]. This was later verified as only the over-expression of p65/p50 but not constitutively active IKK2 could rescue pancreatic cancer cells from the effects of GSK-3β inhibition [126]. It was further demonstrated that GSK-3β inhibition or down-regulation leads to a decrease in NF-κB activity within glioma cell lines [127] and that GSK-3β has a role in modulating cell proliferation in prostate and colon cancer [128,129]. Interestingly it seems that GSK-3β does not affect the nuclear accumulation of NF-κB and can additionally influence NF-κB activity through epigenetic mechanism as it seems to inhibit the NF-κB complex from binding to certain target promoters through histone methylation [130]. The exact molecular mechanism of GSK-3β mediated NF-κB modulation remain, however, elusive and requires further clarification.

Other kinases that have a well-documented link to the NF-κB pathway are various members of the large mitogen-activated protein kinase family (MAPK) including Jun-N-terminal kinase, JNK, and p38 [131]. Both kinases are also triggered by stimuli that activate NF-κB (such as TNFα), as adapter proteins lead to a branching of the signaling towards different downstream pathways. The mutual influences of these kinases and NF-κB are pleiotropic. p38 and related kinases are known to be cofactors in NF-κB activation [55], whereas there is a rather counteracting relationship between NF-κB and JNK [132,133]. Further members of the kinase family, which activate or regulate NF-κB include protein kinase C (PKC) [134,135] and Akt triggered by PI3K [136]. However, it is important to note that the effect of a signaling molecule on NF-κB often strictly depends on the cell type or the micro-environment and that even opposite effects can occur in distinct cell types. This has been reported for instance for the influence of Akt on NF-κB, which is activating in cell types such as epithelial cells, but can be inhibitory in macrophages [137-140].

In addition to adapter molecules, kinases or ubiquitinases and de-ubiquitinases, other classes of molecules were reported to have an influence on NF-κB activity: These include reactive oxygen species (ROS), which are compounds containing free electrons usually linked to oxygen atoms that are not part of an atomic bond. These compounds react quickly with many other substances leading to their oxidation and they can even react with nitrogen dissolved in the aqueous environment generating so called reactive nitrogen species (RNS), which themselves lead to nitrosylations [141]. ROS are often generated within inflammatory environments by the action of neutrophils, which secrete this reactive class of compounds as a defense mechanism against pathogens by a process called “oxidative burst” [142]. Furthermore, ROS occur as a consequence of mitochondrial dysfunction in the course of ischemia/reperfusion events for instance after myocardial infarcts or in the case of transplanted organs, which have been separated from the blood supply for a while followed by reperfusion and sudden availability of oxygen. ROS can activate NF-κB by various mechanisms – and on the other hand, they are also regulated by NF-κB. Several target genes of NF-κB are involved in the de-toxification of ROS but some of them that can also have a pro-oxidant function such as nitric oxid synthases (NOS) pointing at a complex interplay between ROS and NF-κB [143].

### Crosstalk between NF-κB and miRNAs

MicroRNAs (miRNAs) are small noncoding, single stranded RNAs that usually bind to the 3′UTR of protein coding mRNAs typically leading to mRNA cleavage. Alternatively, they can also cause translational repression of their respective targets [144]. Single miRNA-species can have multiple target genes thereby regulating several signaling molecules or pathways. Furthermore, miRNAs are themselves transcriptional targets, thus providing a mechanism for down-regulation of genes by activation of transcription factors. Several miRNAs have been shown to be transcriptional targets of NF-κB including miR-9, miR-21, miR-143, miR-146 and miR-224 [145-150]. These miRNAs are often involved in feedback mechanisms that fine-tune the activity of NF-κB by targeting some of the upstream signaling molecules or members of the NF-κB family themselves. Furthermore, NF-κB can induce the synthesis of proteins that regulate miRNAs. The most important example for that so far is the NF-κB-dependent induction of Lin28, a protein which inhibits the processing and maturation of let-7 miRNAs – a family of miRNAs that is often down-regulated in cancer and which seems to act as tumor suppressor. Let-7 miRNAs target IL-6 – thus a reduction of let-7 leads to higher levels of IL-6 and further activation of NF-κB generating a positive feedback loop [151]. In addition to regulating miRNAs directly or indirectly, NF-κB activity itself is regulated by several miRNAs that either repress NF-κB family members directly or some of the upstream signaling molecules.

**Figure 5:**
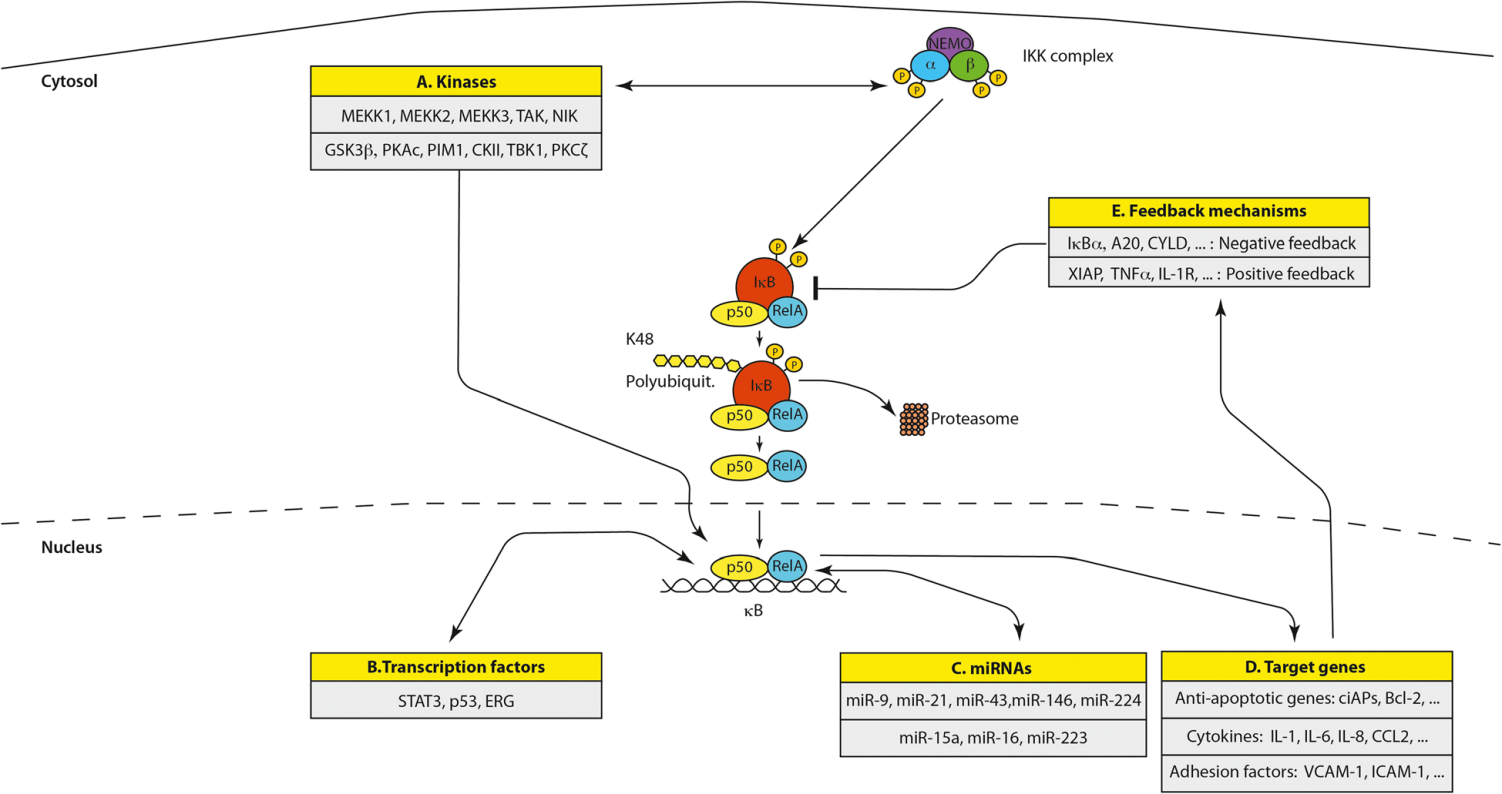
**Crosstalk of the canonical NF-κB pathway with other signaling processes. (A)** Many different kinases can phosphorylate and activate the IKKα and IKKβ subunits of the IKK complex or can enhance NF-κB transcriptional activity. Important examples are glycogen synthase kinase 3β (GSK3β), Protein Kinase B (PKB or Akt), Protein Kinase R (PKR), Protein Kinase C (PKC), Mitogen-Activated Type 3-Protein Kinase 7 (MAP3K7 or TAK1), p38 MAP Kinases or c-Jun N-terminal kinases (JNKs). **(B)** Various transcription factors such as p53, Ets Related Gene (ERG) or Signal Transducer and Activator of Transcription 3 (STAT3) can influence the transcriptional activity of NF-κB or directly activate transcription of NF-κB target genes. **(C)** microRNAs (miRNAs) can be target genes of the NF-κB signaling pathways or can affect the expression of NF-κB family members or effector molecules of the NF-κB activation pathway. **(D)** Prominent target genes of the NF-κB signaling pathway include anti-apoptotic genes as the Baculoviral IAP repeat-containing proteins (BIRCs or cIAPs) and the B-cell lymphoma 2 gene (Bcl-2), cytokines such as Interleukin-1 (IL-1), IL-6, IL-8 and chemokine (C-C motif) ligand 2 (CCL2), adhesion factors including the Vascular Cell Adhesion Molecule 1 (VCAM-1) and the Intercellular Cell Adhesion Molecule 1 (ICAM-1). **(E)** Another layer of complexity of NF-κB signaling are positive and negative feedback mechanism. Examples for positive feedback molecules are the X-linked inhibitor of apoptosis protein (XIAP) as well as TNFα or IL-1. Important negative feedback circuits are generated by the NF-κB target genes IκBα, Cylindromatosis (CYLD) or A20.

In a recent report it was shown that miR-15a, miR-16 and miR-223 can influence IKK1 protein expression during macrophage differentiation. Interestingly, these miRNAs did not affect IKK2 or NEMO expression suggesting that they might be modifiers of the non-canonical NF-κB signaling pathway [152]. Another study showed that miR-502e acts as tumor suppressor by modifying cell proliferation in hepatoma cell lines and hepatocellular carcinoma. The authors suggested that this function is due to the ability of targeting NIK thus directly influencing the non-canonical NF-κB pathway [153]. A depiction of the crosstalk between miRNAs and members of the NF-κB signaling pathway is summarized very well in [146] (Figure 5).

### NF-κB as target in drug combination therapies of cancer

Given its role in the initiation and progression of cancer, the NF-κB signaling pathway is a potent node of pharmacological interference in the clinics (see accompanying article). Since NF-κB is also an essential player in the immune response against cancer, there had always been a reluctance to use NF-κB inhibitors in the treatment of malignancies. Nevertheless, combining classical chemotherapeutics with inhibitors of NF-κB activation seems to result in promising synergies (see [154-165] for some examples). Most cancer drugs are cytotoxic agents that drive proliferating cells into apoptosis, e.g. by interfering with DNA synthesis. Elevated NF-κB activity in cancer cells provides a survival mechanism by up-regulating anti-apoptotic genes, thereby representing a major causative factor for drug resistance [155,157,160,164,166]. Inhibition of NF-κB is also thought to be at least one mechanism of action of proteasome inhibitors in cancer treatment as activation of NF-κB requires the proteasomal degradation of IκB molecules [158,165,167].

A crucial aspect in using NF-κB inhibitors might be an appropriate timing with respect to the cancer stage or the treatment phase. While inhibition of NF-κB is undesirable during the tumor-eliminating phase of the immune system, when immune cells target transformed cells, NF-κB inhibition is expected to have positive effects in the chronic inflammatory phase of tumor progression. The use of NF-κB inhibitors might be beneficial for instance, in the treatment of metastatic prostate cancer when applied synchronously with androgen antagonists or drugs that block androgen synthesis. In this case, the withdrawal of androgens induces the apoptosis of many but often not all androgen-dependent prostate cancer cells. The survival of a small fraction of cells – e.g. due to elevated NF-κB activity and increased anti-apoptosis mechanisms can then result in the development of androgen-independent cancer cells, which are difficult to target. The application of NF-κB inhibitors in combination with anti-androgen therapy is expected to result in a more efficient killing of the prostate cancer cells and a slower or less likely recurrence of cancer. Similar considerations apply to the combination of radiotherapy with inhibition of NF-κB, as radiation-induced up-regulation of NF-κB is thought to counteract the therapy by promoting the survival of cancer cells [168]. Another important aspect is that inhibition of NF-κB might target cancer not only directly by blocking anti-apoptosis mechanisms of malignant cells, but also indirectly by shifting macrophages from the tumor-tolerating M2-polarization stage towards the tumor-attacking M1-stage [102].

## Conclusion

NF-κB represents a central factor in inflammation, stress response, cell differentiation or proliferation as well as cell death. It can be activated by a great variety of stimuli and a complex network of signaling pathways, which can also influence each other. Furthermore, it regulates a huge variety of target genes generating sophisticated feedback circuits that comprise all elements of cellular regulators such as cytokines, growth factors, adhesion molecules, intracellular signaling molecules, transcription factors as well as miRNAs. Thus NF-κB and members of its signaling network have essential roles in the complete flux of biological information from transcription to regulation of RNA-function and turnover, the synthesis of proteins, their functions and their degradation.

## Abbreviations

3′UTR: 3′ untranslated region; ANK: Ankyrin; ATF: Activating transcription factor; ATM: Ataxia telangiectasia mutated; BAFFR: B-cell activation factor; BCL: B-cell lymphoma; BIRC: Baculoviral IAP repeat-containing proteins; CaMKIV: Calmodulin-dependent protein kinase; CBP: CREB-Binding Protein; CC: coiled-coil domain; CHK1: Checkpoint kinase 1; CKII: Casein kinase II; CSC: Cancer stem cell; CXCL: Chemokine ligand; CXR: Chemokine receptor; CYLD: Cylindromatosis; DD: Death domain; DEN: Diethylnitrosamine; DUB: De-ubiquitinase; EGFR: Epidermal growth factor receptor; ERG: Ets Related Gene; GSK: Glycogen synthase kinase; HDAC: Histone deacytlase; HLH: Helix-loop-helix domain; ICAM: Intercellular cell adhesion molecule 1; IKK: IκB kinase; IL: Interleukin; IL-1R: Interleukin-1 receptor; IL-1β: Interleukin-1 β; IκB: Nuclear factor of kappa light polypeptide gene enhancer in B-cells inhibitor; JNK: Jun-N-terminal kinase; LPS: Lipopolysaccharides; LTβR: Lymphtoxin β-receptor; LZ: Leucin-zipper-like motif; MAPK: Mitogen-activated protein kinase; miRNA: MicroRNA; MMP: Matrix metalloproteinase; MSK1: Mitogen- and stress-activated protein kinase; NBD: NEMO-binding domain; NEMO: NF-κB Essential Modulator; NF-κB: Nuclear factor ’kappa-light-chain-enhancer’ of activated B-cells; NIK: NF-κB inducing kinase; PI3K: Phosphatidyl-inositol-3-kinase; PIM1: Proto-oncogene serine/threonine-protein kinase PIM1; PK: Protein kinase; PKAc: Protein kinase A catalytic subunit; PKCζ: Protein kinase Cζ; RANK: Receptor activator for nuclear factor kappa B; RHD: Rel homology domain; RNS: Reactive nitrogen species; ROS: Reactive oxygen species; RSK1: Ribosomal s6 kinase; STAT: Signal transducer and activator of transcription; TA: Transactivation domain; TAK: TGF- β –activating kinase; TAM: Tumor-associated macrophage; TBK1: TANK-binding kinase 1; TLR: Toll-like receptor; TMPRSS2: Transmembrane protease, serine 2; TNFR: Tumor necrosis factor receptor; TNFα: Tumor necrosis factor α; TNPO1: Transportin 1; TRAF: TNF receptor associated factors; VCAM: Vascular cell adhesion molecule; VEGF: Vascular endothelial growth factor; XIAP: X-linked inhibitor of apoptosis protein; ZF: Zinc-finger domain.

## Competing interest

The authors declare no conflict of interest.

## Authors’ contribution

BH and JS wrote the manuscript. Both authors read and approved the final manuscript.

## Authors’ information

BH is a postdoctoral fellow at the Medical University Vienna, Austria. His research focuses on crosstalk and cooperativity of NF-κB signaling with other signaling pathways with an emphasis on prostate cancer. JS is associate professor at the Medical University Vienna, Austria. His research focuses on aspects of inflammation with a focus on NF-κB signaling and the crosstalk with other signaling pathways.
